# Case report: a case of intractable Meniere's disease treated with autogenic training

**DOI:** 10.1186/1751-0759-2-3

**Published:** 2008-01-25

**Authors:** Fumiyuki Goto, Kimiko Nakai, Takanobu Kunihiro, Kaoru Ogawa

**Affiliations:** 1 Department of Otorhinolaryngology, Hino Municipal Hospital, Tamadaira 4-3-1, Hino-shi, Tokyo 191-0062, Japan; 2 Department of Otolaryngology, Keio School of Medicine, Shinanomachi 35, Shinjuku-ku, Tokyo 160-8520, Japan

## Abstract

**Background:**

Psychological stress plays an important role in the onset and course of Meniere's disease. Surgical therapy and intratympanic gentamicin treatment are options for cases that are intractable to conventional medical therapy. Psychotherapy, however, including autogenic training (AT), which can be used for general relaxation, is not widely accepted. This paper describes the successful administration of AT in a subject suffering from intractable Meniere's disease.

**Case presentation:**

A 51-year-old male patient has suffered from fluctuating right sensorineural hearing loss with vertigo since 1994. In May 2002, he was first admitted to our hospital due to a severe vertigo attack accompanied by right sensorineural hearing loss. Spontaneous nystagmus toward the right side was observed. Since April 2004, he has experienced vertigo spells with right-sided tinnitus a few times per month that are intractable to conventional medical therapy. After four months, tympanic tube insertion was preformed in the right tympanic membrane. Intratympanic injection of dexamethasone was ineffective. He refused Meniett therapy and intratympanic gentamicin injection. In addition to his vertigo spells, he suffered from insomnia, tinnitus, and anxiety. Tranquilizers such as benzodiazepines and antidepressants such as serotonin selective re-uptake inhibitors (SSRIs) failed to stop the vertigo and only slightly improved his insomnia. In December 2006, the patient began psychological counseling with a psychotherapist. After brief psychological counseling along with cognitive behavior therapy (CBT), he began AT. He diligently and regularly continued his AT training in his home according to a written timetable. His insomnia, tinnitus, and vertigo spells disappeared within a few weeks after only four psychotherapy sessions. In order to master the six standard formulas of AT, he underwent two more sessions. Thereafter, he underwent follow-up for 9 months with no additional treatment. He is now free from drugs, including tranquilizers, and has continued AT. No additional treatment was performed. When we examined him **six **and nine months later for follow-up, he was free of vertigo and insomnia.

**Conclusion:**

AT together with CBT can be a viable and palatable treatment option for Meniere's disease patients who are not responsive to other therapies.

## Background

Psychological stress plays an important role in the onset and course of Meniere's disease [1]. Surgical therapy and intratympanic gentamicin treatment are options for cases that are intractable to conventional medical therapy. However, psychotherapy including autogenic training (AT) and cognitive behavior therapy (CBT), which can be used for general relaxation and to influence disturbed emotions, is not widely accepted. Only a limited number of reports exist concerning the application of AT and behavior therapy to patients with vertigo [2]. The present paper describes the successful administration of AT together with CBT to a subject suffering from Meniere's disease intractable to several conventional therapies. Written informed consent was obtained from the patient for this publication.

## Case presentation

**Figure 1:**
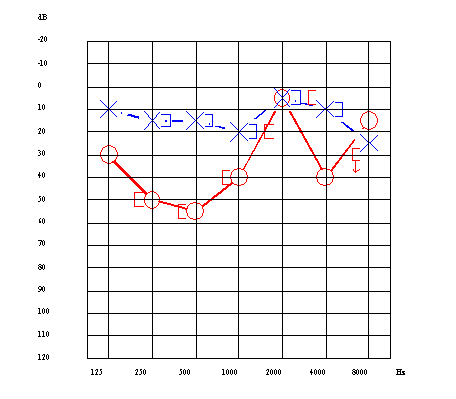
○ together with continuous line and 〔 indicate hearing level of air conduction, and bone conduction in right ear respectively. × together with dotted line and  〕 indicate hearing level of air conduction, and bone conduction in left ear respectively.

**Figure 2:**
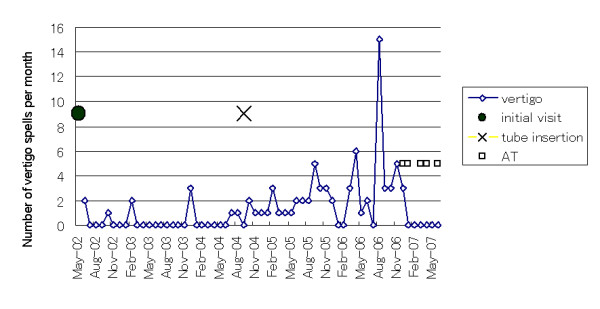
○ together with continuous line and 〔 indicate hearing level of air conduction, and bone conduction in right ear respectively. × together with dotted line and  〕 indicate hearing level of air conduction, and bone conduction in left ear respectively.

**Figure 3:**
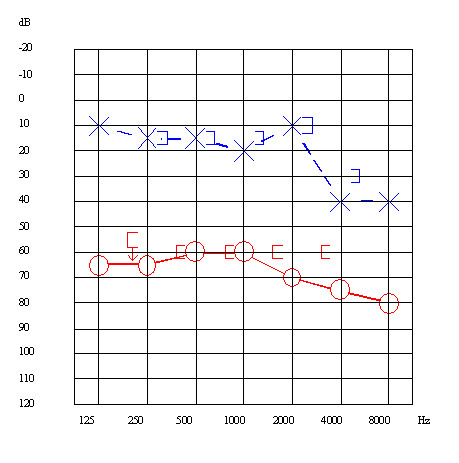
Audiogram on October 2006.

A 51-year-old male patient was first admitted to our hospital on May 2002 because of a severe vertigo attack accompanied by right sensorineural hearing loss. This patient had suffered from fluctuating right sensorineural hearing loss with vertigo since 1994. Audiogram revealed a severe sensorineural hearing loss at 35.0 dB, with a predominance of low frequency impairment in the right ear (Figure 1). The vertigo improved with conventional steroid injections given for one week, but hearing loss did not improve. Thereafter, oral betahistine, adenosine triphosphate disodium (ATP), and isosorbide were prescribed, and vertigo disappeared. Since April 2004, however, a few times per month the patient has experienced vertigo spells that were intractable to conventional medical therapy (Figure 2). Head CT, MRI, and MRA were normal. After four months, we inserted a tympanic ventilation tube into the right tympanic membrane. His vertigo did not improve in the following 15 months. In June 2006, the patient received intratympanic injection of dexamethasone three times within **six **weeks. Dexamethasone treatment, however, was not effective. An audiogram performed in October 2006 revealed that the patient's right-side hearing level deteriorated to 62.5 dB (Figure 3). We recommended alternative therapies including Meniett therapy and intratympanic gentamicin injection; however, he refused.

In addition to vertigo spells, the patient suffered from insomnia, tinnitus, and anxiety. Tranquilizers such as benzodiazepine and antidepressants such as serotonin selective re-uptake inhibitors (SSRIs) did not appreciably alleviate these symptoms. Brotizolam (0.25 mg) slighted ameliorated the insomnia.

Since conventional medical therapy failed to improve his symptoms, we referred him to our psychologist for psychological evaluation and therapy. Although the patient initially declined our referral, he eventually complied, and on December 2006, he began psychological counseling with a psychotherapist. The results of the psychological examination were as follows: Self-rating Depression Scale (SDS), 51; State-Trait Anxiety Inventory (STAI) – Trait Anxiety, 64 (IV); STAI – State Anxiety, 57 (V); Japanese version of the Cornell Medical Index (CMI), IV; Yatabe-Guilford personality test (Y-G), type E. These results indicated that only slight depression was present, but a high degree of anxiety was confirmed. We decided to focus on treating the patient's anxiety, which was addressed by the psychologist. Psychotherapy consisted of one 45-minute session every three weeks. The first session began with a brief introduction to general background information about the cognitive approach, after which the patient was instructed on how to perform AT. The patient performed AT in a relaxed sitting position on a chair for 10 minutes three times a day. No self-monitoring was used. The patient was instructed to perform slow and deep abdominal breathing at the beginning of AT and regular breathing during AT. He diligently and regularly continued this AT routine three times a day in his home according to a written timetable. Astonishingly, on the same day the patient began AT, his insomnia disappeared completely. His insomnia, tinnitus, and vertigo disappeared in a few weeks. He learned all six standard formulas of AT in six psychotherapy sessions. After only four psychotherapy sessions, the patient was free from drugs, including tranquilizers. No additional treatment was performed. When we examined him six and nine months later for follow-up, the patient was free from vertigo and insomnia.

## Conclusion

We present a case of intractable Meniere's disease treated with AT. The substantial efficacy of AT was confirmed in this patient. As of yet, psychotherapy including AT is not widely accepted in the field of otolaryngology.

AT was developed by the German psychiatrist Johannes Schultz and can be achieved by daily self-training sessions of 10 to 15 minutes[3]. AT is a technique for influencing one's autonomic nervous system and it can be used to alleviate many stress-induced psychosomatic disorders. Schultz emphasized parallels between AT and yoga and meditation. AT has been widely applied as a relaxation technique and has been viewed as a very effective way to control pain and to reduce drug dependence substantially [4]. Illnesses that respond well to AT are hypertension [5], tension-type headache [6], and a variety of pain [7].

We used a psychological approach to treat this patient because he refused alternative therapy, such as Meniett and intratympanic injection of gentamicin, and because all other medical therapies failed to ameliorate his symptoms. Before introducing psychotherapy, physicians should acknowledge that some patients may refuse psychotherapy. Indication of psychotherapy should be evaluated carefully. In addition, there are only a limited number of institutes capable of providing psychotherapy to patients with otologic disorders. We now consider that patients who have Meniere's disease with the following characteristics are candidates for psychotherapy [8]: (1) Meniere's disease with severe vertigo spells that are intractable to conventional therapy; (2) patients with insomnia; (3) patients that have an irregular lifestyle; (4) patients with a high degree of anxiety; (5) patients that have a variety of complaints in addition to vertigo spells; and (6) patients in whom it is believed that vertigo is triggered by stress. However, AT is not recommended for patients with low-level anxiety, those with little motivation, or those who lack the intellectual capacity to understand and perform AT. AT can be a viable and palatable treatment option for a patient with Meniere's disease that has not been responsive to other therapies.

## Authors' contributions

FG and KN took part in the treatment of the patient and drafted the manuscript. TK and KO provided instruction and advice on the treatment strategy.
